# Supplementary material for: Phosphoprotein Detection in Sweat Realized by Intercalation Structure 2D@3D g-C3N4@Fe3O4 Wearable Sensitive Motif
Source: Biosensors (Basel). 2022 May 24;12(6):361. doi: 10.3390/bios12060361 (PMC9220892; doi:10.3390/bios12060361)
Supplement: Supplementary file 1 [file biosensors-12-00361-s001.zip › biosensors-1738034-Supporting Information.pdf]

Supporting Information

Phosphoprotein Detection in Sweat Realized by Intercalation Structure 2D@3D g-C_3N_4@Fe_3O_4 Wearable Sensitive Motif

Yuting Qiao[1‡], Lijuan Qiao[2‡], Peize Zhao[1], Peng Zhang[1], Fanbin Wu[1], Jiahui Zhang[1], Li Gao[1*], Bingxin Liu[1*], and Lei Zhang[3]

1 School of Mechanical Engineering, Qinghai University, Xining, 810016, PR China; hiyutingqqq@126.com (Y.Q.); 18733006311@163.com (P.Z.); zhangpeng@qhu.edu.cn (P.Z.); wfb632292561@126.com (F.W.); z276475670@126.com (J.Z.)

2 Research Center of Basic Medical Science, Medical College, Qinghai University, Xining, 810016, PR China; 2014980007@qhu.edu.cn (L.Q.)

3 Department of Mechanical Engineering, University of Alaska Fairbanks, PO Box 755905, Fairbanks, Alaska 99775-5905, United States; lzhang14@alaska.edu (L.Z.)

‡ These authors contributed equally to this work.

* Correspondence: liubx408@nenu.edu.cn (B.L.); 2007990030@qhu.edu.cn (L.G.); Fax: +86-9715310440.

1. EXPERIMENTAL PROCEDURES

1.1 Materials and Methods

Melamine ($C_3H_6N_6$, 99.0%), urea (CH_4N_2O, 99.0%), potassium ferricyanide ($K_3[Fe(CN)_6]$, 99.5%), potassium ferrocyanide ($K_4[Fe(CN)_6]$, 98.0%), ferric chloride hexahydrate ($FeCl_3 \cdot 6H_2O$, 99.0%), ferrous sulfate heptahydratewas ($FeSO_4 \cdot 7H_2O$, 99.0%), ammonium bicarbonate (NH_4HCO_3, 99%), 6-mercapto-1-hexanol (MCH, 98%), potassium chloride (KCl, 99.5%), Tween-20, bovine serum albumin (BSA), Nafion 117 Solution (5%), chloroauric acid ($HAuCl_4 \cdot 4H_2O$, 99.9%), glacial acetic acid (CH_3COOH, 99.9%), ammonia ($NH_3 \cdot H_2O$), sodium dodecyl sulfate (SDS, 98%), β-Casein, α-Lactalbumin, β-Lactoglobulin, dithiothreitol (DTT, 99%), Tris/HCl buffer, iodoacetamide (IAM, 98%), formic acid (CH_2O_2, 99%), acetonitrile (C_2H_3N, 99%), 5X Loading buffer, SDS-PAGE and Brilliant Blue R (AR) were purchased from Aladdin (Shanghai, China). Absolute ethanol was purchased from Sinopharm Chemical ReagentCo (Shanghai, China). Trypsin was purchased from Promega. BCA Protein Assay Kit was purchased from Bio-Rad. All chemical reagents were analytical-grade and directly used without further refinement.

1.2 Characterization

Q Exactive Mass Spectrometer (Thermo Scientific), EASY-nLC 1000 Liquid Chromatography (Thermo Fisher Scientific) , C18 Cartridge (Empore™ SPE Cartridges C18 (standard density), bed I.D. 7 mm, volume 3 mL, Sigma), C18 reversed-phase analytical column (Thermo Scientific Easy Column, 10 cm long, 75 μm inner diameter, 3 μm resin, Sigma), and protein purification reverse phase column (Thermo Scientific Acclaim PepMap100, 100 μm*2 cm, nanoViper C18) were further used to complete sweat proteomics firmness.

The materials appearance images were obtained by scanning electron microscope (SEM, JSM-7900F, JEOL Japan) and transmission electron microscope (TEM, JEM-2100FE, JEOL Japan). The crystal structural analysis of materials was used by Bruker D8-Advance X-Ray Diffractometer (XRD, Bruker D8-Advance). Functional group composition of the materials was observed by Nicolet 6700 Fourier Transform Infrared Spectrometer (FT-IR, Thermofisher). The surface composition was examined by XPS (XPS, Krato-ultra DLD, Shimazduo). Thermogravimetric measurements were carried out with synchronous thermal analyzer (STA449) at 5 °C/min in the air. Hysteresis curves were recorded on vibrating

sample magnetometer (LakeShore 7404). The zeta potential of composite materials was used to determine the surface charge and measured by the malvern Nano Particle Size Analyzer (ZS-90, Malvern Instruments, UK). Nitrogen adsorption specific surface area and pore size were collected on Automatic specific surface and pore size distribution analyzer (Autosorb-iQ2). Absorbance of the materials in the β-Casein solution at 280nm was examined by UV-visible spectrophotometer (SP-754). Cyclic voltammetry (CV), Electrochemical impedance spectroscopy (EIS) measurements, differential pulse voltammetry (DPV) were taken by electrochemistry workstation (CHI660-E) and using Ag/AgCl electrode, platinum wire electrode, and glassy carbon electrode as the reference electrode, the counter electrode and the working electrode with surface modification. The sensor array was constructed by desktop homogenizing mechanism (KW-4A) and real-time testing was taken by portable electrochemical workstation (MEC-VS1).

1.3 Protein Extraction and Peptide Digestion

The proteins in samples (actual human sweat samples) were extracted by SDT (4%(w/v) SDS, 100 mM Tris/HCl pH 7.6, 0.1 M DTT) lysis method. The protein was then quantified using the BCA method.Protein digestion by trypsin was performed according to filter-aided sample preparation (FASP) procedure described by Matthias Mann. The digest peptides of each sample were desalted on C18 Cartridges (Empore™ SPE Cartridges C18 (standard density), bed I.D. 7 mm, volume 3 mL, Sigma). After concentrating by vacuum centrifugation and reconstituting in 40 μL of 0.1% (v/v) formic acid to perform peptide quantification (OD280).

1.4 Filter-aided Sample Preparation Procedure

200 μg of proteins from each sweat sample were incorporated into 30 μL SDT buffer (4% SDS, 100 mM DTT, 150 mM Tris-HCl pH 8.0). The detergents, DTT and other low molecular weight components were removed by repeated ultrafiltration (Microcon units, 10 kD) using UA buffer (8 M urea, 150 mM Tris-HCl pH 8.0). 100 μL of iodoacetamide (100 mM IAA in UA buffer) was then added to block reduced cysteine residues while the samples were incubated in the dark for 30 min. The filters were first washed three times with 100 μL of UA buffer and then twice with 100 μL of 25 mM NH_4HCO_3 buffer. Finally, the protein suspensions was added to 40 μL of 25mM NH_4HCO_3 buffer with 4 μg of trypsin (Promega) was digested overnight at 37 °C, and the resulting peptides were collected as a filtrate. The peptides from each sweat sample were desalted on C18 Cartridges (Empore™ SPE

Cartridges C18 (standard density), bed I.D. 7 mm, volume 3 mL, Sigma), concentrated by vacuum centrifugation and reconstituted in 40 µL of 0.1% (v /v) formic acid. The peptide content was estimated from UV spectral density at 280 nm, using a 0.1% (g/l) solution with an extinction coefficient of 1.1, calculated from the frequencies of tryptophan and tyrosine in vertebrate proteins.

1.5 SDS-PAGE

A 12.5% SDS-PAGE gel was prepared in the previous stage, and the protein of each sample with a loading amount of 20 µg was mixed with 5X Loading buffer and boiled for 5 minutes and electrophoresed at 14 mA for 90 minutes. After electrophoresis, protein bands were observed by Coomassie blue R-250 staining.

1.6 LC-MS/MS Analysis

LC-MS/MS data were analyzed on Q Exactive Mass Spectrometer (Thermo Scientific). The mass spectrometer was coupled with the Easy nLC liquid chromatography (Thermo Fisher Scientific) for 120 minutes. The protein purification reverse phase column (Thermo Scientific Acclaim PepMap100, 100 µm*2 cm, nanoViper C18) was loaded by loading the peptides and connected to a C18 reversed-phase analytical column (Thermo Scientific Easy Column, 10 cm long, 75 µm inner diameter, 3 µm resin) containing buffer solution A (0.1% Formic acid). In addition, the separation was performed using a linear gradient of buffer B (84% acetonitrile and 0.1% Formic acid) at a flow rate of 300nl/min under IntelliFlow technology control. The mass spectrometer was operated in positive ion mode. The MS data were acquired by using a data-dependent top10 method to dynamically selecting the most abundant precursor ions from the full spectrum scan (300–1800 m/z) for HCD fragmentation. Regarding the automatic gain control (AGC) target setting of 3e6, the maximum injection time is 10 ms. Dynamic exclusion duration was 40.0s. Survey scans were acquired at 200 m/z with a resolution of 70,000, followed by setting the resolution of the HCD spectrum to 17,500 at 200 m/z and the isolation width to 2 m/z. The normalized collision energy was 30 eV, and the underfill was defined as 0.1%. The instrument was run with peptide recognition mode enabled.

1.7 Identification and Quantitation of Proteins

The MS raw data integration for each sample was merged and searched for identification and quantification using MaxQuant 1.5.3.17 software. The relevant parameters and descriptions of the software are as follows:

Table S1. MaxQuant Identification and Quantification Parameters Indexes.

Items	Values
Enzyme	Trypsin
Max Missed Cleavages	2
Fixed Modifications	Carbamidomethyl (C)
Variable Modifications	Oxidation (M)
Main Search	6 ppm
First Search	20 ppm
MS/MS Tolerance	20 ppm
Database	xxxx<br>for example, uniprot_mouse_76417<br>represent: “uniprot”, public database (http://www.uniprot.org/); “mouse”, organism species; “76417”, the number of sequences
Database Pattern	Reverse
Include Contaminants	True
Protein FDR	≤0.01
Peptide FDR	≤0.01
Peptides used for Protein Quantification	Use razor and unique peptides
Time Window (match between runs)	2min
Protein Quantification	LFQ
min. ratio count	1

Notes: Intensity-based absolute quantification (iBAQ) and LFQ are two different methods for protein quantification provided by Maxquant software. iBAQ Intensity reveals the level of protein expression in the sample X based on iBAQ algorithm，which is approximation to the absolute concentration of the protein in the sample. LFQ Intensity reveals the level of protein expression in the sample X based on LFQ algorithm，which is often used in the comparison between groups.

1.8 Cluster analysis of Phosphorylated Peptides

Cluster 3.0 (http://bonsai.hgc.jp/~mdehoon/software/cluster/software.htm) and Java Treeview software (http://jtreeview.sourceforge.net) were used to performing hierarchical clustering analysis. Euclidean distance algorithm for similarity measure and average linkage clustering algorithm (clustering uses the centroids of the observations) for clustering were selected when performing hierarchical clustering. A heat map was often presented as a visual aid in addition to the dendrogram.

1.9 Preparation of g-C_3N_4@Fe_3O_4

The preparation method of g-C_3N_4@Fe_3O_4 was added 0.07 mol of urea in 50 mL of deionized water (DW) and dissolve it, and added 0.02 mol of melamine and stir for 30 minutes. The mixture was hydrothermally reacted at 180 °C for 24 h. The intermediate was washed with DW and absolute ethanol and dried in a vacuum oven at 60 °C for 12 h. The intermediate was heated to 520 °C within 40 minutes and calcined in the muffle furnace for 4 h to obtain a yellow solid (g-C_3N_4). Then 0.4 g of g-C_3N_4 was added to 200 mL of DW and 200 mL of absolute ethanol (V_{DW}:$V_{ethanol}$=1:1), and ultrasonically dispersed for 2 h to form a uniform yellow solution. In addition, the orange iron salt solution was mixed with 0.4 g $FeSO_4{\cdot}7H_2O$ and 0.2919 g $FeCl_3{\cdot}6H_2O$ in 80 mL of deionized water, dissolved in ultrasound for 30 minutes and poured into the above solution. The above-mentioned mixed solution was heated in a water bath at 80°C for 10 minutes, added with 1.5 mol/L NaOH solution to adjust the pH to 10, and reacted with magnetic stirring for 60 minutes to form a brown-black solution. The composite product was taken out by magnet decantation, washed with deionized water and ethanol, and dried in vacuum at 60°C for 12 hours to obtain g-C_3N_4@Fe_3O_4 composite, named CNFeO-0.4.

Under other conditions unchanged, composite with different ratios were prepared by adding samples with masses of 0.2 g, 0.3 g, 0.6 g, and 1.2 g g-C_3N_4 for characterization and comparison, which were named CNFeO-0.2, CNFeO-0.3, CNFeO-0.6, CNFeO-1.2. In addition, the ferroferric oxide (Fe_3O_4) was prepared according to the above conditions without adding g-C_3N_4.

The preparation method of pure g-C_3N_4 was obtained by directly calcining melamine as the raw material and heating at 13° C/min at 520° C for 4 hours.

1.10 Phosphoprotein Adsorption Assay

The phosphoprotein adsorption kinetics process was to configure a β-Casein protein solution with a concentration gradient of 0.1 mg/mL-1.0 mg/mL through an ammonium bicarbonate buffer solution with a concentration of 50 mM and a pH of 8.0 and add edg-C_3N_4@Fe_3O_4 composite material to shake evenly at 4 °C for adsorption. The adsorption capacity of phosphoprotein under different pH conditions was achieved by changing the buffer solution pH to 6.0, 6.5, 7.0, 7.5, 8.5, 9.0 and adsorbing for 3 hours. The adsorption isotherm model and adsorption isotherm were studied at different β-Casein concentrations for 3 hours.

After the adsorption is completed, the centrifugation step was carried out in a high-speed refrigerated centrifuge. The centrifugal speed was set to 5000 rpm and the centrifugal time was set to 15 min. The supernatant was taken out and the ultraviolet absorbance was directly measured at 280 nm using a UV-visible spectrophotometer. During the experimental test, three parallel experiments were performed, and the average value was taken to investigate the stability, error, and material stability of the experiment.

The adsorption capacity of β-Casein for g-C_3N_4@Fe_3O_4 composite materials under different adsorption conditions could be calculated by formula conversion which refer to (1). Among them, C_0 and C_1 represent the concentration of β-Casein protein solution before and after adsorption, respectively. V_0 and M represent the added volume of β-Casein protein solution and the added mass of g-C_3N_4@Fe_3O_4 composite, respectively. And Q represents the amount of β-Casein adsorbed by the g-C_3N_4@Fe_3O_4 composite material. The Freundlich adsorption isotherm model is calculated and described by the Freundlich adsorption isotherm equation which refer to (2). Among them, Q_e and C_e represented the adsorption capacity of the composite material and the concentration of β-Casein at the adsorption equilibrium, respectively. In addition, K_i and n represent the adsorption equilibrium constant, respectively.

$$Q=\frac{(C_0\text{-}C_1)\times V_0}{m}\times 1000 \tag{1}$$

$$Q_e=K_iC_e^{\frac{1}{n}} \tag{2}$$

1.11 Preparation of Electrode

The bare glassy carbon electrode with a diameter of 3 mm was polished with 1, 0.3, and 0.05 μm alumina powder in sequence, rinsed thoroughly with deionized water and absolute ethanol, and dried. The electrochemical three-electrode system used glassy carbon electrode

as working electrode, Ag/AgCl electrode as reference electrode and platinum wire electrode as counter electrode, supplemented with 5 mM $K_3[Fe(CN)_6]/K_4[Fe(CN)_6]$ and 0.1 M KCl solution as electrolyte solution for electrochemical detection. Before the electrochemical test, the three electrodes were tested by CV at a potential range of -0.4 V to 0.6 V in the electrolyte solution and a scanning speed of 100 mV/s. Until a stable redox signal appears on the screen, the working electrode could be considered as pretreatment. After the process was completed, it can be modified in the next step.

1.12 Preparation of Work Electrode for Sensing

5 mg of the synthesized composite was dissolved in a mixed solution containing 500 μL of DW and 500 μL of ethanol, and then the liquid was homogenized by sonication for 1 h. The glassy carbon electrode was immersed in a sealed electrolytic cell containing a 10 mM chloroauric acid solution. In the sealed electrolytic cell, nitrogen was passed to remove oxygen for 10 min, and constant potential deposition was carried out at a voltage of -0.2 V to obtain a gold nanoparticle-modified electrode Au/GCE [1]-[3]. The g-C_3N_4@Fe_3O_4/Au/GCE electrode was obtained by measuring 10 μL of 5 mg/mL g-C_3N_4@Fe_3O_4 composite on the Au/GCE mirror. The MCH/g-C_3N_4@Fe_3O_4/Au/GCE electrode was obtained by dripping 10 μL of 1 mM MCH blocking solution on the mirror surface, waiting for sealing for 90 min and washing with deionized water. The β-Casein/MCH/g-C_3N_4@Fe_3O_4/Au/GCE electrode was obtained by dripping 10 μL 1 mg/mL β-Casein on the MCH/g-C_3N_4@Fe_3O_4/Au/GCE electrode mirror surface and waiting for adsorption for 180 min. Before the final electrochemical test, it was need 0.02% Tween-20 solution and deionized water to clean and then the electrode to dry.

[1] Manikandan, S.; Durairaj, S.; Boateng, E.; Sidhureddy, B.; Chen, A. Electrochemical Detection of Nitrite Based on Co_3O_4-Au Nanocomposites for Food Quality Control. J*ournal of The Electrochemical Society* **2021**, 168, 107505.

[2] Cui'e, Z.; Bei, Y.; Duan, B.; Jin, W.; Shu, L.; Ping, Y.; Cai, W.; Yukihide, S.; Yukou, D. Electrochemical synthesis of gold nanoparticles decorated flower-like graphene for high sensitivity detection of nitrite. *Journal of Colloid and Interface Science* **2017**, *488*, 135-141.

[3] Mousavi-Majd, A.; Ghasemi, S.; Hosseini, R. Zeolitic imidazolate framework derived porous ZnO/Co_3O_4 incorporated with gold nanoparticles as ternary nanohybrid for determination of hydrazine. *Journal of Alloys and Compounds* **2022**, *896*, 162922.

1.13 Preparation of Sensing Array Construction

First, the PI flexible substrate three electrodes were ultrasonically cleaned in deionized water for 20 minutes, then washed with water, and air-dried naturally. Then taken 25 mg of the g-C_3N_4@Fe_3O_4 composite and dispersed it in 5 mL of solution (V_{DW}:$V_{ethanol}$:V_{Nafion}=10:9:1) ultrasonically dispersed uniformly. Second, spin-coated onto the substrate at room temperature at a speed of 2000 rpm for 60 seconds, and naturally dried to ensure the completion of the sensor array construction.

1.14 DFT Computation Methods

The calculation in this paper adopts the first-principles molecular dynamics calculation method based on density functional theory (DFT), and the calculation software was VASP [1]-[2]. The exchange-correlation potential was approximated by a generalized gradient in the form of Perdew-Wang91 [3]. The projected plus plane wave (PAW) method was chosen to describe the interaction between electrons and ions [4]. The cutoff energy of a plane wave was 400 eV. All calculations were taken spin into account. The energy convergence criterion between two electron steps is 10^{-4} eV. In the surface calculation, a supercell of p (5 × 4) was used to sample the Brillouin zone with a K grid centered on a Γ point of size 1 × 1 × 1. Atomic coordinates were optimized until the maximum force per atom was not greater than 0.01 eVÅ^{-1}. To avoid interactions between adjacent periodic structures, a vacuum layer over 45 Å is established in the c-direction. The convergence criterion adopted for the adsorption of protein molecules is 0.05 eVÅ^{-1}. The first-principles molecular dynamics calculations in this paper were investigated using the NVT ensemble, with a calculation time step of 1 fs and a total simulation time of 100 ps.

[1] Kresse, G.; Furthmüller, J. Efficiency of ab-initio total energy calculations for metals and semiconductors using a plane-wave basis set-ScienceDirect," in *Computational Materials Science* **1996**, *6*, 15-50.

[2] Kresse G.; Hafner, J. Ab initio molecular dynamics for liquid metals. *Phys Rev B Condens Matter* **1993**, *g48*, 13115-13118.

[3] Perdew, J. P.; Wang, Y. Accurate and simple analytic representation of the electron-gas correlation energy. *Physical review. B* **1992**, *45*, 13244-13249.

[4] Blöchl, P. E. “Projector Augmented-Wave Method. *Physical Review B* **1994**, *50*, 17953-17979.

2. SWEATOMICS ANALYSIS

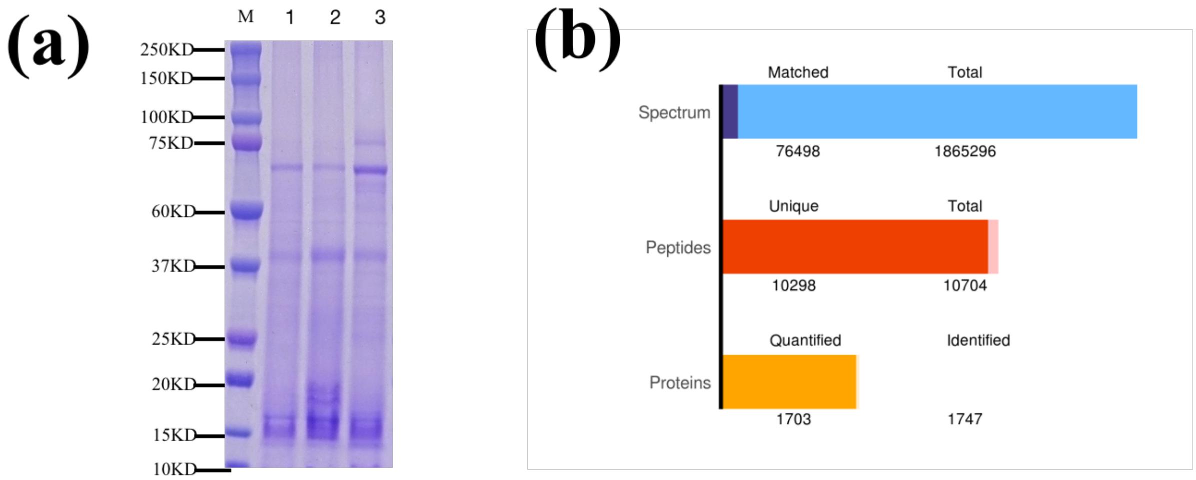

Figure S1. (a) SDS-PAGE detection of protein samples from human sweat, and (b) Statistical histogram of identification and quantification results.

2.1 Sweat Proteomics

The SDS-PAGE bands of human sweat sample protein samples determined by Bradford quantification were clear, in which rubber particle proteins exhibited uneven distribution. Among them, the protein with molecular weight less than 25 kD has a higher content, and the band difference between each sample was obvious, which can be used for subsequent experimental steps.

2.2 Overall Information Statistics of the Proteome

After analysis by MaxQuant 1.5.3.17 software, the number of matched secondary spectra (Total Spectrum) was 1,865,296, and the number of matched spectra (Matched Spectrum) in the database was 76,498. The number of identified peptides (Peptides) was 10704, and 16298 unique peptide sequences (Unique Peptide) were identified.

Among them, a total of 109 phosphoproteins including glycogen phosphorylase, 2,3-cyclic-nucleotide 3-phosphodiesterase, and tartrate-resistant acid phosphatase type 5 phosphoproteins were identified, and they were associated with different physiological states such as carbohydrate metabolism, RNA metabolism in myelin cells and Hawking disease, respectively.

A total of 1747 identified proteins (Identified Proteins) and 1703 quantified proteins (Quantified Proteins) were identified. Among them, a total of 109 phosphoproteins were identified, and the relevant information through data retrieval is shown in Table. S8.

3. MATERIAL CHARACTERIZATION

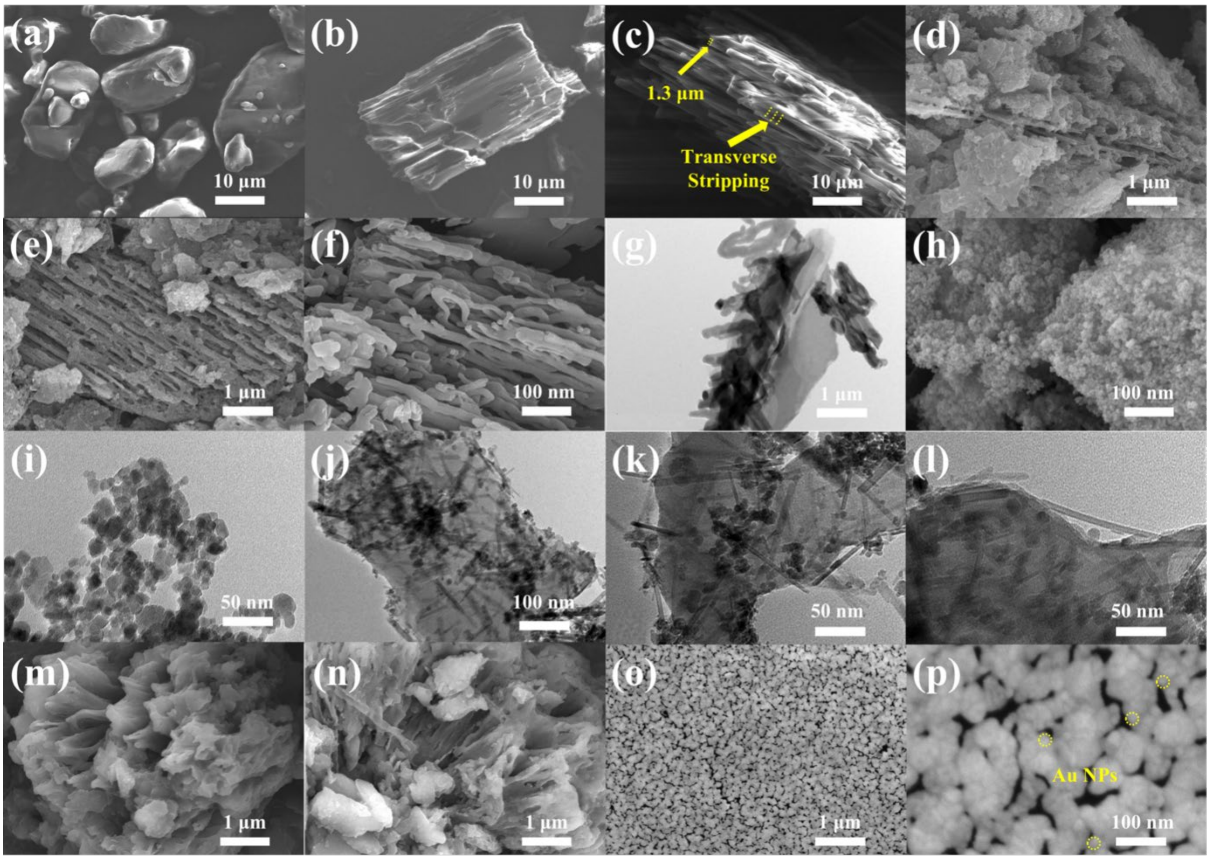

Figure S2. The SEM images of (a) melamine, (b) urea, and (c) intermediate. The g-C_3N_4 SEM images of (d), (e), (f) and (g) at different viewing angles and the g-C_3N_4 TEM images. The Fe_3O_4 SEM images of (h) and TEM images of (i). The g-C_3N_4@ Fe_3O_4 SEM images of (j), (k), and (l). The SEM images of pure g-C_3N_4 of (m) and (n). The SEM images of Au NPs of (o) and (p).

3.1 Morphology Analysis

Figure. S2d to S2f also showed the morphologies of g-C_3N_4 at more angles. Among them, Figure S2d showed that g-C_3N_4 has many nanosheets, clearly interspersed with some vertically aligned nanotubes. After further observation, Figure. S2e showed that many regularly arranged nanotube structures are interspersed between the nanosheets in an orderly manner, forming g-C_3N_4 with a sheet-tube-sheet intercalation structure. Figure. S2f presented the nanotube-like structure at a larger scanning magnification. It can be seen that many nanotubes were ordered and oriented, and the tube-like structure was clear and obvious.

In addition, Figure. S2h showed Fe_3O_4 image under SEM, and it can be seen that many nanoparticles aggregate to form a cluster distribution. However, the particle morphology on

the surface could not be observed carefully because the nanoparticle size was too small. Therefore, the effective formation of many Fe_3O_4 nanoparticles can be further seen through the TEM in Figure. S2i. Likewise, it can be seen from Figure. S2i that many Fe_3O_4 nanoparticles were uniformly distributed.

Figure. S2j to S2l showed the TEM under different nanosheet layers in g-C_3N_4@ Fe_3O_4. Nanosheets of different sizes as well as nanotubes and nanowires also supported the nanoparticle mosaic, further demonstrating the microscopic morphology of the intercalated structure.

Figure. S2m to S2n showed the SEM of the synthesis of without urea doped g-C_3N_4 by direct calcination of melamine.Figure. S2m showed the g-C_3N_4 synthesized directly by high-temperature thermal polymerization from melamine. Obviously, pure g-C_3N_4 exhibited a typical multilayer structure with irregular volume. In addition, no voids could be found by observation. Likewise, as shown in Figure. S2n, pure g-C_3N_4 showed a distinct stacking structure of nanosheets.

Figure. 2o to 2p presented the electrochemically deposited gold nanoparticle (Au NPs) layer morphologies at different scan magnifications. The formation of the Au NPs layer can be seen from Figure.2o, which could be significantly increase the specific surface area of the electrode surface. Figure. 2p showed the morphology of the gold nanoparticle layer at high magnification. It can be seen that many Au NPs are aggregated into clusters and dispersed uniformly, which can effectively increased the dispersibility of the electrode surface.

3.2 TG/DSC

When the temperature was from room temperature to 120°C, the slight fluctuation in weight loss could be attributed to the evaporation of a small part of water vapor in the sample.

In the whole heating process, the mass loss of Fe_3O_4 was about 0.13%, indicating that it has good thermal stability. In g-C_3N_4, three mass loss occurred as a whole in thermal decomposition. Terminated at 580.1°C, 694.1°C and 724.9°C, the sample loss were approximately 19.98%, 25.61% and 56.68%, respectively. After 724.9°C, g-C_3N_4 was completely burned and there was obvious weight loss, and the final residual mass of g-C_3N_4 nanomaterial was about 3.74%.

The thermal decomposition of CNFeO-0.4 first occurred in the 425.4°C to 425.4°C, which could be attributed to oxidation and decomposition in the air. The second weak thermal decomposition was terminated at 756.8°C, and a large amount of decomposition was completed while accompanied by an exothermic reaction. When the temperature reached 756.8°C, the g-C_3N_4 in the composite was completely burnt. The remaining residue could be judged to be Fe_3O_4, which accounts for 41.47% of the mass fraction. The results showed that the thermal stability of CNFeO-0.4 was significantly higher than that of g-C_3N_4, but it was still lower than that of Fe_3O_4. The reason was that there was a certain strong interaction between the interfaces of the composite. It could be analyzed that the protection and barrier effect of the high temperature resistant Fe_3O_4 on the g-C_3N_4 would limited the mobility of the g-C_3N_4 or delayed the thermal decomposition reaction. In addition, the poor flow of gas between the g-C_3N_4 structures prevented the thermal decomposition of g-C_3N_4, increased the energy required for thermal decomposition and leads to improved heat resistance of composite [1]-[2].

[1] Yin, Z.; Ping, Z.; Sai, Z.; Li, K.; Zhi, Y.; Hong, Q.; Si, L. Preparation and properties of magnetic separation Fe_3O_4/g-C_3N_4 composites. *Acta Materiae Composite Sinica* **2018**, *35*, 3189-3195.

[2] Li, X.; Zhang, L.; Wang, X.; Yu, Q. PANI/g-C_3N_4 composites synthesized by interfacial polymerization and their thermal stability and photocatalytic activity. *Acta PhysicoChimica Sinica* **2015**, *31*, 764-770.

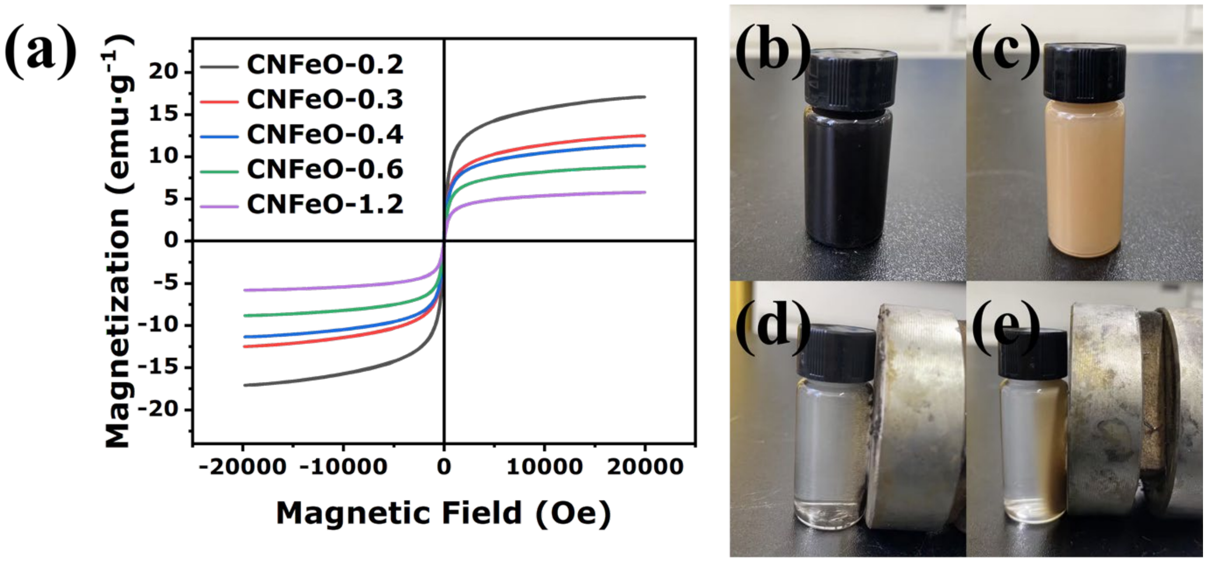

Figure S3. The VSM patterns of (a) different ratio g-C_3N_4@ Fe_3O_4 composite. The optical picture of (b) Fe_3O_4, (c)CNFeO-0.4 dispersed in deionized water and (d) Fe_3O_4, (e)CNFeO-0.4 under an external magnetic field.

3.3 VSM

The remanence and coercivity of all magnetic nanomaterials tended to zero, and they were generally superparamagnetic. The saturation magnetization of Fe_3O_4 measured at room temperature was 71.67 emu/g, indicated that it has strong ferromagnetism [1]. With the increase of g-C_3N_4 doping, Figure. S3A exhibited the saturation magnetization of the composite gradually decreases. The reason was that the wrapping and blocking effect of g-C_3N_4 on Fe_3O_4 [2]. In addition, the saturation magnetization corresponding to CNFeO-0.2, CNFeO-0.3, CNFeO-0.4, CNFeO-0.6 and CNFeO-1.2 are 17.03 emu/g, 12.47 emu/g, 11.33 emu/g, 8.81 emu/g and 5.77 emu/g, respectively.

As shown in Figure. S2b to S2e, the optical picture showed that the Fe_3O_4 and CNFeO-0.4 composite were uniformly dispersed in the DW, and both were obviously concentrated in the direction of the magnet under an external magnetic field.

[1] Zhang, X.; Ren, B.; Li, X.; Liu, B.; Jiang, G. High-efficiency removal of tetracycline by carbon-bridge-doped g-C_3N_4/Fe_3O_4 magnetic heterogeneous catalyst through photo-Fenton process. *Journal of Hazardous Materials* **2021**, *418*, 126333.

[2] Zhi, Z.; Xu, T.; Wen, F.; Zhi, L.; Peng, H.; Tian, W.; Yong, Y.; Chun, L. Studying of Co-doped g-C_3N_4 and modified with Fe_3O_4 quantum dots on removing tetracycline. *Journal of Alloys and Compounds* ***2018***, *775*, 248-258.

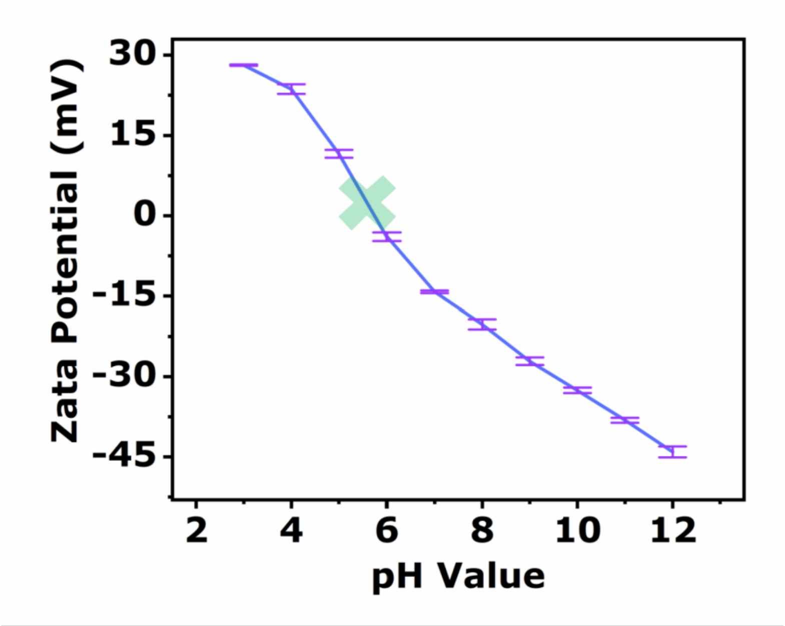

Figure S4. Zeta Potential of CNFeO-0.4.

3.4 Zeta

The electrical properties of the surface of the material sample determined its electrical properties under various pH conditions [1]. The Zeta potential of the CNFeO-0.4 under different pH conditions was shown in Figure. S4. The isoelectric point of the composite was pH=5.747. It showed that when the pH of the solution is less than 5.747, the surface of the material showed a positive charge distribution. When the pH of the solution was more than 5.747, the surface of the material showed a negative charge distribution.

[1] Zhang, J.; Lin, C.; Han, C.; Su, Y. Adsorption Properties of Magnetic Magnetite Nanoparticle for Coexistent Cr (VI) and Cu (II) in Mixed Solution. *Water* **2020**, *12*, 446.

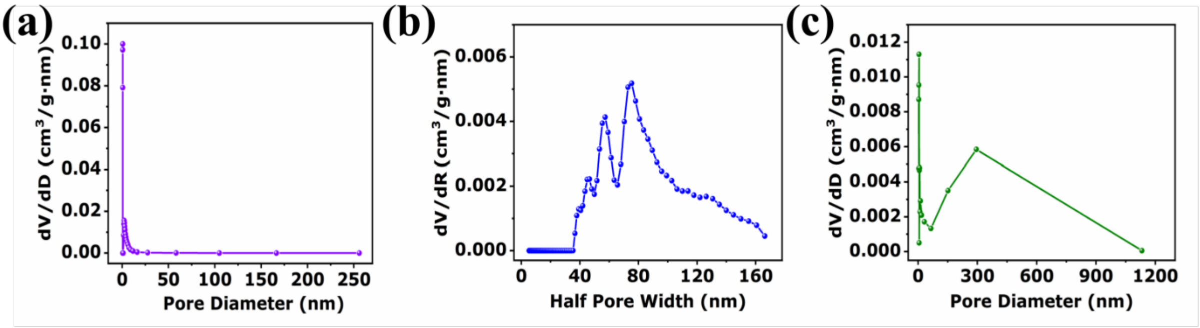

Figure S5. (a) pore diameter of g-C_3N_4, (b) half pore width of Fe_3O_4 and (c) pore diameter of CNFeO-0.4.

3.5 Nitrogen Adsorption and Desorption

In Figure. 3f and Figure. S5a, g-C_3N_4 showed a nonporous adsorption isotherm type. Its specific surface area, pore diameter and pore volume were 53.338 m²/g, 2.250 nm, and 0.065 cc/g, respectively.

As shown in Figure. 3f, Fe_3O_4 and CNFeO-0.4 adsorption-desorption isotherm had an obvious H3 type hysteresis loop IV isotherm, showed a typical reversible adsorption trend, indicated that the sample surface has a mesoporous structure [1]. The specific surface area calculated by the BET model is 72.621 m²/g and 85.895 m²/g, respectively. The adsorption capacity of the composite jumped when the P/P_0 was in the range of 0.9-1.0, which was caused by the accumulation of Fe_3O_4 to form mesopores and lamellar inclusions of g-C_3N_4. The results showed that a larger specific surface area composite was synthesized [2]. Due to the aggregation of plate-like particles or the combination of slit-like pores, the adsorption capacity was greatly increase. The Fe_3O_4 formed between g-C_3N_4 flakes make the degree of exfoliation of the flakes more dispersed, and the specific surface area of the flakes were increased. It can provide more active sites, which is conducive to promoting the transmission capacity of electrons [3].

In addition, The half-pore width of Fe_3O_4 in Figure. S5b was 75.341 Å, and the pore size and pore volume of CNFeO-0.4 in Figure. S5c were 55.069nm and 1.652 cc/g, respectively.

[1] Li, P.; Wang, Y.; Wang, J.; Dong, L.; Fan, Q. Carboxyl groups on g-C_3N_4 for boosting the photocatalytic U(VI) reduction in the presence of carbonates. *Chemical Engineering Journal* **2021**, *414*, 128810.

[2] Thommes, M. Physical Adsorption Characterization of Nanoporous Materials. *Chemie Ingenieur Technik* **2010**, *82*, 1059-1073.

[3] Yu, X.; Jing, Z.; Ping, W.; Zhao, T.; Jun, Z.;Yan, H.; Nuzahat, H. Nuzahat Habibul. Removal of U(VI) from aqueous solution via photocatalytic reduction over WO_3/g-C_3N_4 composite under visible light. *Chemical Engineering Journal* **2022**, 428, 131209.

4. PERFORMANCE ANALYSIS

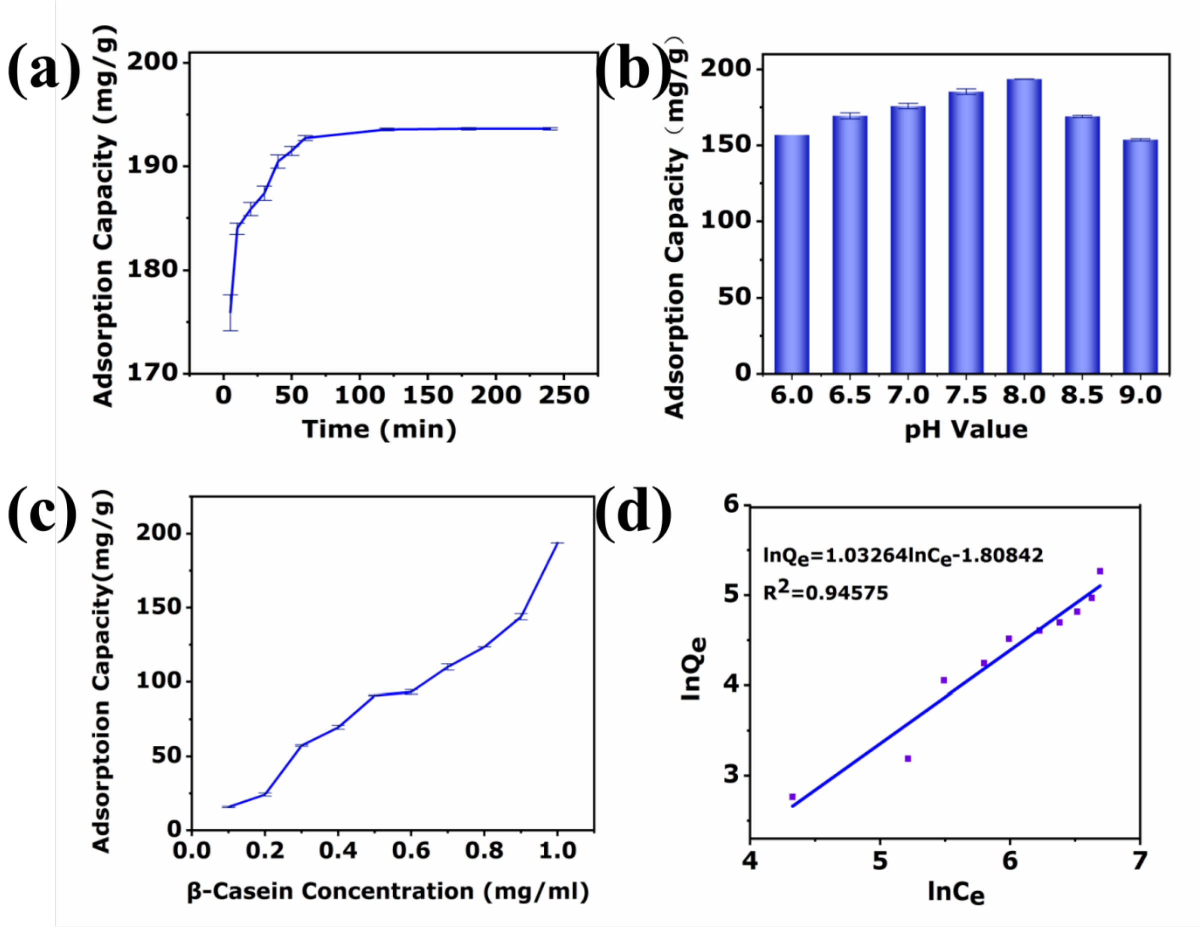

Figure S6. (a) Adsorption kinetics curve of β-Casein by CNFeO-0.4, (b) Adsorption capacity of CNFeO-0.4 for β-Casein at different pH values, (c) Freundlich isothermal adsorption model of CNFeO-0.4 for β-Casein, and (d) Adsorption isotherm study of CNFeO-0.4 for β-Casein.

The experimental results of adsorption kinetics were shown in Figure. S6a. The adsorption process could be divided into fast adsorption and stable adsorption. At the beginning, the adsorption kinetics curve showed a gradual increase trend with the extension of the adsorption time, and the slope of the curve was larger. The adsorption amount increased faster with the extension of the adsorption time within 1 h. The corresponding adsorption capacities at 5 min, 10 min, 20 min, 30 min, 40 min, 50 min, and 1h were 175.9 mg/g, 184 mg/g, 185.87 mg/g, 187.4 mg/g, 190.47 mg/g, 191.5 mg/g and 192.73 mg/g. The reason was that the distribution density of protein molecules in the solution is larger, and the large specific surface area of the composite provides more group sites that can react with the protein. They might be electrostatically attracted to promote specific binding. The adsorption capacity increased slowly after 1h. As the adsorption time is extended to 3 h to 4h, the slope

of the kinetic curve tended to zero, and the adsorption amount tended to gently reach a state of adsorption equilibrium. Among them, the corresponding adsorption capacities of 2 h, 3 h and 4 h are 193.57 mg/g, 193.63 mg/g, and 193.63 mg/g, respectively. The reason was that the β-Casein protein is a macromolecular substance, and the surface of the composite material is gradually covered by protein molecules. In addition, the gradual decreased of the protein concentration in the solution leaded to a decrease in the molecular distribution density. The degree of contact of groups on the surface of the material was reduced. Considering the influence of adsorption kinetics, the adsorption time was selected as 3 h, which paves the way for the subsequent electrochemical test of sensor performance.

Figure. S6b showed the phosphoprotein adsorption capacity under different pH buffers. As the pH value of the buffer solution increased, the degree of adsorption capacity presented a significant change trend. The isoelectric point of β-Casein was about 4.6 [1], but the adsorption capacity at pH=8.0 reached the maximum of 193.63 mg/g. The reason was that the pH of β-Casein is 8.0 as the node, and the phosphate in the solution should be predicted to exist in the form of HPO_4^{2-} at this time

$Q_{8.0}$=193.63mg/g > $Q_{7.5}$=185.37mg/g > $Q_{7.0}$=175.83mg/g > $Q_{6.5}$=169.37mg/g > $Q_{8.5}$=169.0mg/g > $Q_{6.0}$=156.8mg/g > $Q_{9.0}$=153.7mg/g. Therefore, the pH value of the buffer solution system has a certain influence on the occupancy of phosphoprotein by the adsorption material.

The Freundlich isotherm adsorption model was shown in Figure. S6c. The adsorption capacity at adsorption equilibrium was plotted against the logarithm of the concentration of β-Casein. The adsorption model equation was obtained by fitting the data: $\ln Q_e = 1.03264 \ln C_e - 1.80842$, $R^2=0.94575$. A linear linear relationship was obtained between $\ln Q_e$ and $\ln C_e$, which verifies that the adsorption behavior of the composite to β-Casein had a good correlation with the Freundlich adsorption isotherm. It could be inferred that the adsorption of β-Casein by the composite is multimolecular layer adsorption, and only one

linear equation curve could be obtained according to the data fitting, indicated that there is a main force combination between the composite and β-Casein.

Figure. S6d presented the adsorption isotherm study. The analysis chart showed that as the protein concentration of the solution increases, the adsorption capacity of the composite increased simultaneously. The reason was that when the concentration of the protein solution is low, the protein molecules could not saturate the surface group sites of the composite. However, the increase of protein concentration and molecular density increased the probability of mutual adsorption and contact between the group sites on the surface of the composite and the protein molecules, which promoted the increase of the overall adsorption capacity and reaches saturation. It could be seen from the Figure. S6d that when the concentration of β-Casein protein is in the range of 0.1 mg/mL to 0.9 mg/mL, the adsorption capacities are respectively 15.77 mg/g, 24.23 mg/g, 57.3 mg/g, 69.6 mg/g, 90.97 mg/g, 93.3 mg/g, 110.0 mg/g, 123.43 mg/g, 143.83 mg/g. The adsorption capacity increase of the composite material to β-Casein under different concentrations showed a obvious dynamic increasing trend. Finally, when the concentration of β-Casein was 1 mg/mL, the adsorption capacity reached 193.63 mg/g.

[1] Han, Z.; Fang, J. Technology of extracting casein from milk in laboratory. *Animal Husbandry and Feed Science* ***2010***, *31*, 83−85.

[2] Fa, X.; Feng, W.; Gui, L.; Yun, M.; Cheng, F.; Huan, W.; Giesy, P. Removal of Phosphate from Eutrophic Lakes through Adsorption by in Situ Formation of Magnesium Hydroxide from Diatomite. *Environmental Science & Technology* ***2014***, *48*, 582-590.

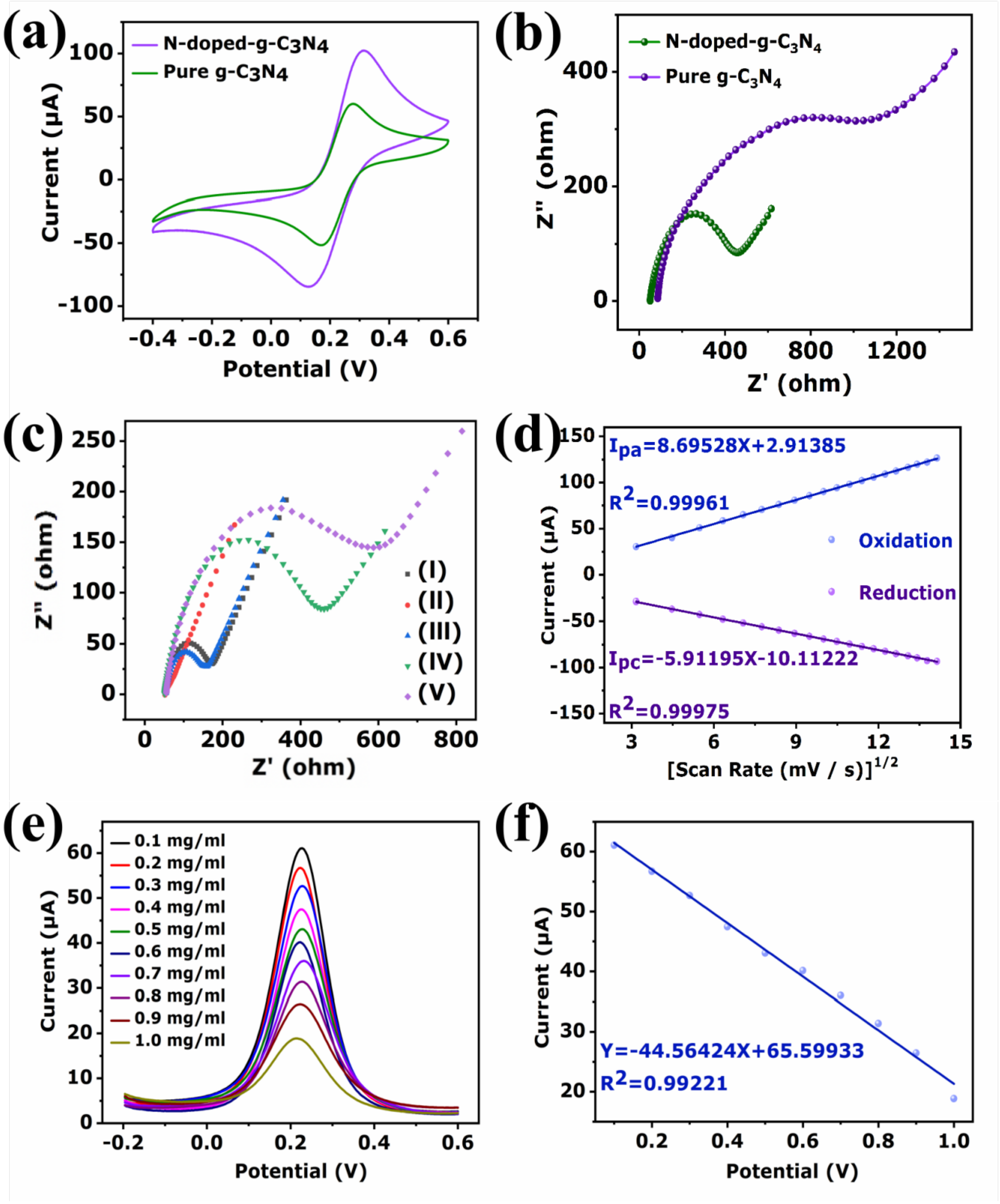

Figure S7. The performance comparison between N-doped-g-C_3N_4 and Pure g-C_3N_4 of (a)CV, and (b)EIS, (c) Enlarged view in EIS of electrodes under different modified conditions, (d) Fitting equations and R^2 for CV in different sweep speeds of 10-200 mV/s, (e) DPV for different concentration of β-Casein (0.1 mg/mL to 1.0 mg/mL), and (f) Fitting equations and R^2 for CV.

Table S2. Comparison of the electrochemical sensing performance of different field.

Sample Solution	Materials	LOD	Liner Range	References
Water	Zn^{II}-DPA[a]	0.22 ppm		[1]
Glioblastoma cell	Silicon photonic microring resonator arrays	0.6 pM	3.55-log	[2]
Food	NH_2-TiO_2/UCNPs[b] -rGO	9.2×10^{-5} mg/mL	0-1 mg/mL	[3]
Electrolyte	DPA-Zn^{2+}[c]		≥1 nM	[4]
Food	NH_2-TiO_2/MUA[d]/ AuE[e]-QCM[f]	0.09 mM	1.0×10^{-3} -1.0 mg /mL	[5]
Cancer cell	Zr-FeTCPP[g]-MOF		0.1–40 nM/40–150 nM	[6]
Electrolyte	DPA-NH_2[h]		≥1 nM	[7]

Sweat	g-C_3N_4@Fe_3O_4	9.7 µM	0.01-1mg/mL	This work

toward phosphoprotein detection.

a: Zinc(II)-dipicolylamine; **b:** Upconversion nanomaterials; **c:** Dipicolylamine–zinc chelates 4; **d:** 11-mercaptoundecanoic acid; **e:** Au electrode; **f:** Quartz crystal microbalance 5; **g:** Fe (III) meso-Tetra (4-carboxyphenyl) porphine 6; **h:** 4-[bis(2-pyridylmethyl)aminomethyl]aniline

[1] Tsukuru, M.; Tsuyoshi, M.;Petr, K.; Pavel, Jr.; Shi, T. Antibody- and Label-Free Phosphoprotein Sensor Device Based on an Organic Transistor. *Anal. Chem*. **2016**, *88*, 1092–1095.

[2] James, W.; Aurora, A.;, Nicholas, V.; Hongi, Y.; Mark, J.; Ryan, B. Rapid, Multiplexed Phosphoprotein Profiling Using Silicon Photonic Sensor Arrays. *ACS Central Science* **2015**, *1*, 374−382.

[3] Jian, G.; Shi, L.; Shuo, W.; Jun, W. Determination of Trace Phosphoprotein in Food Based on Fluorescent Probe-Triggered Target-Induced Quench by Electrochemiluminescence. *Journal of Agricultural and Food Chemistry* **2020**, *68*, 12738-12748.

[4] Saima, N,; Mubarak, Al.; Ishtiaq, A.; Christof, N.; Wolfgang, E. Biomolecular Detection with a Single Nanofluidic Diode Decorated with Metal Chelates. *ChemPlusChem* **2020**, *85*, 101002.

[5] Jian, G.; Guo, F.; Shuo, W.; Jun, W. Quartz crystal microbalance sensor based on 11-mercaptoundecanoic acid self-assembly and amidated nano-titanium film for selective and ultrafast detection of phosphoproteins in food. *Food Chemistry* **2021**, *344*, 128656.

[6] Xin, L.; Shuang, E.; Xu, C. Metal-organic framework/3,30,5,50-tetramethylbenzidine based multidimensional spectral array platform for sensitive discrimination of protein phosphorylation. *Journal of Colloid and Interface Science* **2021**, *602* , 513–519.

[7] Saima, N.; Mubarak, A.; Ishtiaq, A.; Christof, N.; Wolfgang, E. Phosphoprotein Detection with a Single Nanofluidic Diode Decorated with Zinc Chelates. *ChemPlusChem* **2020**, 85, 587–594

Table S3. The chemical bond change of β-Casein on g-C_3N_4@Fe_3O_4 with different components.

Chemical Bonds in β-Casein	Bond Length (g-C_3N_4/β-Casein)	Bond Length (Fe_3O_4/β-Casein)	Bond Length (g-C_3N_4@Fe_3O_4 /β-Casein)
C-N	1.45Å	1.46Å	1.47Å
C-C	1.54Å	1.54Å	1.57Å
C-H	1.12Å	1.15Å	1.17Å
C-O	1.42Å	1.41Å	1.46Å
O-H	0.99Å	0.97Å	1.02Å
N-H	1.03Å	1.03Å	1.07Å
C-S	1.84Å	1.85Å	1.89Å

Table S4. The chemical bond change of β-Casein on g-C_3N_4.

Chemical Bonds in g-C_3N_4	Bond Length (g-C_3N_4/β-Casein)
C-N (Internal)	1.32Å
N-H (Interface)	1.04Å

Table S5. The chemical bond change of β-Casein on Fe_3O_4.

Chemical Bonds in Fe_3O_4	Bond Length (Fe_3O_4/β-Casein)
Fe-O (Internal)	2.01Å
O-Fe (Interface)	2.33Å

Table S6. The chemical bond change of β-Casein on g-C_3N_4@Fe_3O_4.

Chemical Bonds in g-C_3N_4@Fe_3O_4	Bond Length (g-C_3N_4@Fe_3O_4/β-Casein)
C-N (Internal)	1.40Å
N-H (Internal)	1.05Å
Fe-O (Internal)	2.04Å
Fe-N (Interface)	2.44Å

Table S7. The adsorption energy for the different components of g-C_3N_4@Fe_3O_4 on β-Casein.

Materials	Ads Energy (eV)
g-C_3N_4	-1.89
Fe_3O_4	-0.92
g-C_3N_4@Fe_3O_4	-2.41
